# The Therapeutic Value of Pulsatilla

**Published:** 1880-05

**Authors:** 


### THE THERAPEUTIC VALUE OF PULSATILLA.

PULSATILLA is rapidly groiwng in favor with many practitioners. Though a very old remedy, having been known to Dioscorides and Pliny, it fell into disuse and was not reinstated till the beginning of the present century.

I have used pulsatilla mainly in simple dysmenorrhœa, where it has proved of decided utility. Its scope is, however, doubtless much wider. A very prominent lawyer of this city told me not long since, that after trying the bromide, valerianates and other remedies of repute for the headache caused by excessive mental application, he found no relief till he made use of the tincture of pulsatilla. He is now never without it, and uses no other medicine for the cure of his headaches, which I know to be very severe. The tincture should be made from the fresh leaves and given with caution. The dose is from 3 to ten drops. —*Dr. Tucker, in the Chicago Medical Gazette.*

